# Seizure-like Symptoms Induced by Hypomagnesemia in a Patient with Incomplete Small Bowel Obstruction; A Case Report

**DOI:** 10.31661/gmj.v13i.3350

**Published:** 2024-05-01

**Authors:** Jiyan Weng, Wenjun Chen, Mingjin Zhu

**Affiliations:** ^1^ Department of Rehabilitation Medicine, Tongde Hospital of Zhejiang Province, Hangzhou, China; ^2^ Department of Pharmacy, Xixi Hospital of Hangzhou, Hangzhou, China

**Keywords:** Hypomagnesemia, Seizure, Intestinal Obstruction, Nutritional Support

## Abstract

Background: Seizure-like symptoms are rare in older patients without brain damage. Small bowel obstruction is a common clinical disorder for older patients that can cause electrolyte disturbances and nutritional disorders. Hypomagnesemia is a frequently overlooked electrolyte disorder. Moreover, magnesium deficiency can lead to severe seizure-like symptoms. Case Report: An 85-year-old man was admitted to the hospital with weakness and slow movement. Shortly after hospitalization, he experienced incomplete small bowel obstruction; thus, parenteral nutrition and intravenous esomeprazole were administered. When intestinal obstruction was relieved, the patient suddenly experienced seizure-like symptoms three times, and 24-h electroencephalogram did not capture any epileptiform pattern. After excluding other causes, we considered serum magnesium deficiency as a diagnosis. Low serum magnesium levels were related to a shortage of absorption due to small bowel obstruction, excess excretion of renal dysfunction, and the use of proton pump inhibitor. However, the exact mechanism underlying the hypomagnesemia-induced seizure-like activity remained unclear. After adjusting the nutritional support and magnesium supplementation, the patient’s serum magnesium level returned to normal, and he was free of seizure-like activity. Conclusion: Hypomagnesemia is often asymptomatic, but it can lead to severe seizure-like symptoms. It is important to pay attention to the serum magnesium level and nutritional intake in patients with an incomplete small bowel obstruction.

## Introduction

Magnesium is one of the major electrolytes with divalent cation in living cells and is a vital element in human physiological functioning [1]. It acts as a cofactor for >300 enzymes [2]. Moreover, magnesium ions (Mg2+) play an essential role in energy production including adenosine triphosphate utilization for various physiological processes [3], particularly in the brain, heart, and skeletal muscles [4]. Magnesium is associated with neuromuscular transmission [2][5]. Serum Mg2+ levels are tightly regulated by the kidneys and intestine. Hypomagnesemia is a relatively common feature in critically ill patients and is associated with poor outcomes; however, it is sometimes overlooked, as it can commonly be asymptomatic until manifested [6]. Magnesium can modulate excitotoxicity-related epilepsy, and hypomagnesaemia can cause seizures [7][8]. In addition, Mg2+ supplementation has been shown to be beneficial in treating pre-eclampsia, eclampsia, and conditions associated with symptomatic seizures [9]. Here, we present a case of hypomagnesemia-induced seizure-like symptoms in an older patient with incomplete intestinal obstruction.

## Case Presentation

**Figure-1:**
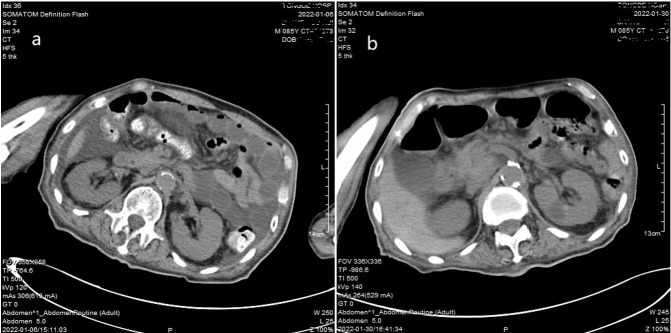
Figure 1. (a) Abdominal computed tomography (CT) showing the fluid-filled dilated small bowel; (b) Abdominal CT showing the dilated loop of small bowel

**Figure-2:**
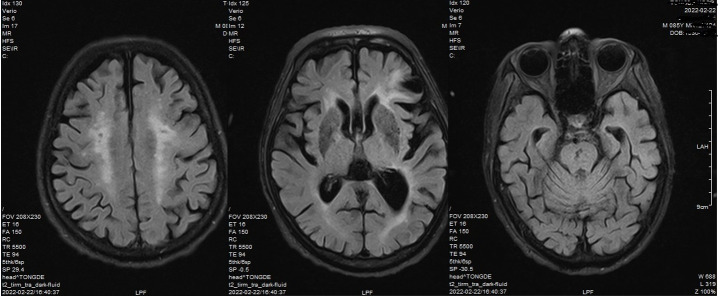
Figure 2. Magnetic resonance image of the brain showing multiple chronic lacunar infarcts

An 85-year-old man was hospitalized for an unstable gait. The patient presented with weakness, poor appetite and slow movement. He had a history of hypertension and transient ischemic attack. Laboratory tests showed that the patient had anemia and hypoproteinemia. He received 75mg aspirin, 20mg atorvastatin, 300mg polysaccharide-iron complex, 150mg irbesartan, and protein powder daily. The patient underwent a magnetic resonance imaging (MRI) of the brain, which showed multiple old lacunar lesions in the bilateral basal ganglia, brain stem and parietal lobe. Balance and strength training was arranged. In early January 2022, the patient experienced abdominal distension and repeated vomiting. Abdominal computed tomography (CT) revealed small intestinal obstruction (Figure-1a). After immediate fasting, gastrointestinal decompression, anti-inflammatory therapy, and parenteral nutritional support, the patient’s condition improved, and he followed a liquid diet 1 week later. However, in late January, the patient experienced projectile vomiting again with severe abdominal pain and distension, along with a lack of flatus or defecation. An indwelling ileus tube was recommended after consultation with the gastroenterologist. However, the patient’s family refused because of reluctance to expose the patient to endoscopy risk and abdominal CT showed that the intestinal obstruction had worsened (Figure-1b). Conservative and supportive treatment included fasting, gastric decompression, enema, parenteral nutrition (Kabiven PI, 1440mL [Fresenius Kabi, Wuxi, China]). Octreotide 200mg was prescribed to reduce gastrointestinal secretion. Moreover, a proton pump inhibitor (PPI) injection, esomeprazole (40mg), was administered for gastro protection. After 2 weeks of treatment, the symptoms of intestinal obstruction were relieved. Peptison (EN suspension [SP]: 500mL, 1 kcal/mL [Nutricia, Wuxi, China]) 500mL quaque die (QD) to bis in die (BID) nasogastric tube feeding was administered as enteral nutrition. On the evening of February 17, the patient experienced sudden limb convulsions with loss of consciousness, shortness of breath, increased heart rate, and decreased oxygen saturation. The epilepsy-like symptoms lasted 3-4 min and then disappeared. During the next 3 days, the patient had two more seizure-like activities. However, the 24-h electroencephalogram still did mot capture the epileptiform pattern. The brain MRI result was similar to that before (Figure-2). The cause of the seizure-like symptoms was unknown. However, we re-checked the patient’s clinical data and found that his serum magnesium and phosphate levels were 0.52 mmol/L and 0.53 mmol/L, respectively (Table-1). As the serum magnesium was deficient, diluted magnesium sulfate (2.5g) was administered intravenously for 2 days, and composite potassium hydrogen phosphate (2mL) was administered orally daily. The enteral nutrition Peptison (SP, 1kcal/mL) was replaced with Nutrison fibre (EN suspension [TPF]: 500 mL, 1.5 kcal/mL [Nutricia, Wuxi, China]) 500mL BID to strengthen the patient’s nutritional status. The patient did not have seizure-like symptoms in the following days and the blood test revealed that the Mg2+ levels had risen to 0.70 mmol/L. During the following month, the patient remained free of seizure-like symptoms as the serum Mg2+ levels were close to the normal range.

## Discussion

**Table:** Table1. Blood Tests of the Patient

Date	Ca ^2+^	Mg ^2+^	P ^5+^	WBC	Hb	hs-CRP	Cr	Urea	ALT	AST
	[2.11-2.52] mmol/L	[0.75-1.02] mmol/L	[0.85-1.51] mmol/L	[3.5-9.5] 10E9/L	[130-175] g/L	[0-8] mg/L	[57-110]μmol/L	[3.6-9.5] mmol/L	[9-50] U/L	[15-40] U/L
2022/1/31	1.80	0.79	0.85	5.50	70.00	17.16	94.00	11.80	6.00	16.00
2022/2/16	1.82	0.52	0.53	-	-	-	148.00	13.90	10.00	28.00
2022/2/22	1.79	0.54	0.40	8.30	65.00	20.20	-	-	-	-
2022/2/24	1.83	0.70	0.67	-	-	-	150.00	20.40	16.00	25.00

**WBC:** white blood cell; **hs-CRP:** high-sensitivity C-reactive protein; **Hb:** hemoglobin; **Cr:** creatinine; **ALT:** Alanine aminotransferase; **AST:** Aspartate aminotransferase

Magnesium, a critical mineral in the human body, is involved in the regulation of several physiological functions. First-level evidence supports the use of magnesium in the prevention of many common health conditions such as migraine headache, asthma, premenstrual syndrome, preeclampsia, various cardiac arrhythmias and metabolic disorders (obesity, diabetes, changes in lipid metabolism, and hypertension) [10][11]. Magnesium has also been considered an adjunct for depression, attention deficit disorder, and kidney stone prevention [12][13]. A cohort study showed that low serum Mg2+ levels are significantly associated with the risk of all-cause mortality in a community-based population [14]. Clinical features of hypomagnesemia include neuromuscular irritability, central nervous system hyperexcitability, and cardiac arrhythmias [15]. Several case reports have shown that hypomagnesemia may contribute to seizures [6][8][16][17]. In the present case, the patient’s seizure disorder was associated with hypomagnesemia. Although the exact mechanism underlying hypomagnesemia-induced seizures remains uncertain, it may be associated with disinhibition of the N-methyl-D-aspartate type glutamate receptor-sodium channel complex [18]. Magnesium is mainly regulated by a balanced between small intestinal absorption and renal excretion. The recommended magnesium intake for adult men and women is 350 mg/day and 300 mg/day, respectively [19]. This patient’s hypomagnesemia was due to digestive system dysfunction and was probably related to renal causes. The patient had incomplete intestinal obstruction, and recurrent vomiting resulted in electrolyte disturbance and mineral absorption dysfunction. Magnesium deficiency in critically ill patients has frequently been overlooked [20]. The parenteral nutrition solution Kabiven contains only 96mg Mg2+, which is well below the required values. As serum creatinine levels increase, patients may experience renal injury. Presumably, tubular dysfunction leads to enhanced urinary magnesium excretion, contributing to hypomagnesemia [21]. After the relief of intestinal obstruction, we chose Peptison nasogastric tube feeding (500ml BID) as enteral nutrition. Peptison is a short peptide that can be easily absorbed and is more likely to improve the nutritional status and immune function, especially in critically ill patients [22]. However, the Mg2+ concentration in Peptison (115mg/500ml) was insufficient. Moreover, the patient had received intravenous esomeprazole 40 mg QD for 1 week. A systematic review revealed that PPIs may increase the risk of hypomagnesemia [23]. Old age and long-term PPI use may increase the likelihood of developing hypomagnesemia [24]. It has been postulated that PPI-induced hypomagnesemia is caused by an increase in luminal pH, which leads to impaired intestinal Mg2+ absorption. This may be due to the reduced Mg2+ solubility in the small intestine or changes in the composition and function of the gut microbiome in the colon [25]. Therefore, esomeprazole use might be one of the causes of hypomagnesemia in the present case; however, compared with other PPIs, esomeprazole has the lowest risk [26]. After we prescribed intravenous magnesium sulfate, replaced Peptison with Nutrison fibre (containing Mg2+ 170mg /500mL) 500mL BID, and discontinued esomeprazole, the patient’s serum magnesium returned to normal, and he was free of seizure-like activity.

## Conclusion

Intestinal obstruction can lead to electrolyte imbalances and magnesium deficiency, often overlooked as a cause of seizures. Kabiven lacks sufficient magnesium, requiring additional supplementation and monitoring. Peptison aids absorption but lacks energy and magnesium, suggesting prompt transition to intact-protein enteral nutrition. Long-term proton pump inhibitor use should be avoided. Seizure-like symptoms, possibly from metabolic/electrolyte issues, require monitoring for hypomagnesemia in patients with intestinal obstruction on PPI therapy.

This work was funded by the Zhejiang Scientific Research Foundation of Traditional Chinese Medicine (2021ZB056), Zhejiang Provincial Medical and Health Science and Technology Program Project (2023RC139).

This study was approved by the Ethics Committees of Tongde Hospital of Zhejiang Province (ZTDLS2002-164-JY).

## Conflict of Interest

All authors declare that they have no conflict of interest.
